# Anomaly Detection in Embryo Development and Morphology Using Medical Computer Vision-Aided Swin Transformer with Boosted Dipper-Throated Optimization Algorithm

**DOI:** 10.3390/bioengineering11101044

**Published:** 2024-10-18

**Authors:** Alanoud Al Mazroa, Mashael Maashi, Yahia Said, Mohammed Maray, Ahmad A. Alzahrani, Abdulwhab Alkharashi, Ali M. Al-Sharafi

**Affiliations:** 1 Department of Information Systems, College of Computer and Information Sciences, Princess Nourah Bint Abdulrahman University (PNU), P.O. Box 84428, Riyadh 11671, Saudi Arabia; asalmazroa@pnu.edu.sa; 2 Department of Software Engineering, College of Computer and Information Sciences, King Saud University, P.O. Box 103786, Riyadh 11543, Saudi Arabia; mmaashi@ksu.edu.sa; 3 Department of Electrical Engineering, College of Engineering, Northern Border University, Arar 91431, Saudi Arabia; 4 Department of Information Systems, College of Computer Science, King Khalid University, Abha 62521, Saudi Arabia; mmarey@kku.edu.sa; 5 Department of Computer Science and Artificial Intelligence, College of Computing, Umm-AlQura University, Makkah 24382, Saudi Arabia; aalzahrani@uqu.edu.sa; 6 Department of Computer Science, College of Computing and Informatics, Saudi Electronic University, Riyadh 11673, Saudi Arabia; aalkharashi@seu.edu.sa; 7 Department of Computer Science and Artificial Intelligence, College of Computing and Information Technology, University of Bisha, Bisha 67714, Saudi Arabia; aalsharafi@ub.edu.sa

**Keywords:** embryo development, computer vision, boosted dipper-throated optimization, swin transformer, image preprocessing

## Abstract

Infertility affects a significant number of humans. A supported reproduction technology was verified to ease infertility problems. In vitro fertilization (IVF) is one of the best choices, and its success relies on the preference for a higher-quality embryo for transmission. These have been normally completed physically by testing embryos in a microscope. The traditional morphological calculation of embryos shows predictable disadvantages, including effort- and time-consuming and expected risks of bias related to individual estimations completed by specific embryologists. Different computer vision (CV) and artificial intelligence (AI) techniques and devices have been recently applied in fertility hospitals to improve efficacy. AI addresses the imitation of intellectual performance and the capability of technologies to simulate cognitive learning, thinking, and problem-solving typically related to humans. Deep learning (DL) and machine learning (ML) are advanced AI algorithms in various fields and are considered the main algorithms for future human assistant technology. This study presents an Embryo Development and Morphology Using a Computer Vision-Aided Swin Transformer with a Boosted Dipper-Throated Optimization (EDMCV-STBDTO) technique. The EDMCV-STBDTO technique aims to accurately and efficiently detect embryo development, which is critical for improving fertility treatments and advancing developmental biology using medical CV techniques. Primarily, the EDMCV-STBDTO method performs image preprocessing using a bilateral filter (BF) model to remove the noise. Next, the swin transformer method is implemented for the feature extraction technique. The EDMCV-STBDTO model employs the variational autoencoder (VAE) method to classify human embryo development. Finally, the hyperparameter selection of the VAE method is implemented using the boosted dipper-throated optimization (BDTO) technique. The efficiency of the EDMCV-STBDTO method is validated by comprehensive studies using a benchmark dataset. The experimental result shows that the EDMCV-STBDTO method performs better than the recent techniques.

## 1. Introduction

Infertility is an increasing problem globally. According to the World Health Organization, one out of every six couples have problems resulting in issues of infertility [1]. Various factors are associated with infertility, possibly containing difficulties like genetic or anatomical problems, sexually transmitted diseases, physiological dysfunction, immunological or endocrinological problems, and much more [2]. Additionally, the growing tendency concerning late pregnancy owing to financial reasons, career concerns, or not finding a good partner increases the requirement for IVF facilities. The fertilization and in vitro embryo growth are based on environmental conditions that must be unchanging and right regarding light, media pH, air quality, temperature, and osmolality. Embryos are classified morphologically by a standardized scoring method, depending upon many parameters like fragmentation percentage, embryo age, size regularity, and blastomere number [3]. In the observation, the embryo culture must be reserved from the incubator, handing over risks of contact with unpredictable surroundings, which may trouble the embryo’s growth. To remove such a risk, a benign and semi-automatic incubator method, named the time-lapse (TL) process, is accepted for IVF, which authorizes a real-time inspection of embryo culture in incubation [4]. The physical explanation of embryo culture generates individual results. It uses a significant amount of effort and time, even though the application of a real-time visual documentation task is in video format. To eliminate such complexity, the embryo valuation future has maintained attention to the growth of an automatic embryo grading approach using TL-based data and even a traditional microscope [5]. CV and different AI models and devices have been applied inside fertility hospitals to improve efficacy. The AI models help embryologists by automating different functions, namely annotation of cell stages, embryo selection, prediction of implanting potential and live-birth results, and scoring and grading of morphological phases [6].

From a more comprehensive perspective, these AI algorithms are classified into two kinds: (1) AI models that examine portions or complete time-lapse videos to detect morphological patterns associated with particular results like pregnancy, and those that (2) examine the present development of morphology to predict the prospects of embryo growth. Many applications are addressed through the theory of image processing and CV. DL is an advanced AI method in various fields and is considered an essential approach to future human-assistant technology [7]. As specified in earlier investigations, DL models are especially convolutional neural networks (CNNs), which generally keep enormous latent spaces for medical image technologies, healthcare, and medicinal diagnosis. Unlike traditional ML and deep neural network (DNN) methods, they streamline the process of feature engineering, present intellectual learning over a hierarchic representation of the data, proficiently handle huge data amounts, and prove their dominance in identifying anomalies in medical images [8]. The growing incidence of infertility emphasizes the urgent requirement for effectual monitoring and intervention techniques in assisted reproductive technologies. As couples increasingly seek IVF due to diverse societal and personal factors, confirming optimal conditions for embryo development becomes significant [9]. Conventional evaluation approaches mainly depend on subjective evaluations, which can result in inconsistencies and potential oversight of critical developmental anomalies. By incorporating advanced CV and ML methods, the accuracy and efficiency of embryo analysis can be improved. This technological shift promises to enhance embryo selection and reduce environmental disturbances during substantial growth phases. Ultimately, employing these innovative models may result in greater success rates in IVF procedures, presenting hope to many couples facing infertility threats [10].

This study presents an Embryo Development and Morphology Using a Computer Vision-Aided Swin Transformer with a Boosted Dipper-Throated Optimization (EDMCV-STBDTO) technique. The EDMCV-STBDTO technique aims to accurately and efficiently detect embryo development and is critical for improving fertility treatments and advancing developmental biology using medical CV techniques. Primarily, the EDMCV-STBDTO method performs image preprocessing using a bilateral filter (BF) model to remove the noise. Next, the swin transformer method is implemented for the feature extraction technique. The EDMCV-STBDTO model employs the variational autoencoder (VAE) method to classify human embryo development. Finally, the hyperparameter selection of the VAE method is implemented using the boosted dipper-throated optimization (BDTO) technique. The efficiency of the EDMCV-STBDTO method is validated by comprehensive studies using the benchmark dataset. The major contribution of the EDMCV-STBDTO method is listed below.

- The EDMCV-STBDTO technique utilizes the *BF* model to efficiently mitigate noise in embryo images, which improves overall image quality. This preprocessing step crucially enhances the reliability of subsequent feature extraction, allowing for more precise classification outcomes in the evaluation of embryo development.
- The ST method is employed by the EDMCV-STBDTO technique to enable advanced feature representation, effectually capturing complex patterns within the embryo image data. This methodology improves the capability of the approach to discern subtle differences in embryo quality, ultimately resulting in an enhanced classification accuracy. The integration of this cutting-edge architecture emphasizes the significance of robust feature extraction in DL applications.
- The EDMCV-STBDTO model employs a VAE method to classify human embryo development, capitalizing on its capacity to learn intrinsic data dispersions. This methodology allows for efficient modeling of the underlying characteristics of embryo images, facilitating precise differentiation between quality classes. By incorporating the VAE, the approach improves the overall predictive performance of the classification task.
- The BDTO model is implemented by the EDMCV-STBDTO technique for the effectual selection of hyperparameters in the VAE method, which improves the performance and accuracy of the approach. This optimization model streamlines the tuning process, allowing for a more efficient exploration of the hyperparameter space. By enhancing the VAE’s configuration, the approach results in improved classification outcomes in embryo quality analysis.
- The incorporation of an ST with a VAE model for embryo classification depicts a novel methodology, integrating advanced DL techniques to substantially improve predictive capabilities in reproductive science. This integration allows for an enhanced feature extraction and representation, effectually addressing intrinsic data patterns in embryo images. By employing these advanced techniques, the model not only enhances classification accuracy but also contributes to a deeper understanding of embryo quality evaluation.

## 2. Literature Review

Liao et al. [11] proposed a medical consent-tractable DL technique called Esava (Embryo Segmentation and Viability Assessment) to quantifiably determine the growth of IVF embryos utilizing optical microscopic images. Utilizing the Quicker R-CNN method as a base, the Esava method was created, advanced, validated, and trained for accurate and healthy blastomere recognition. A new method, Crowd-NMS, was presented and applied in Esava to improve object recognition and accurately measure embryonic cells and their extent consistency. Sharma et al. [12] proposed to develop the domain of ART by using AI to measure embryos at the morula phase. The morula phase is vital, and the possibility of identification is lower in the cleavage phase in embryo development. This technique traverses the present gap in considering the morula morphological structure and possibly converts the aided reproductive processes by enhancing the selection criteria of the embryo. Yang et al. [13] proposed ML and time-lapse microscopy to interpret variations in embryonal development dynamic with maternal ageing. The author studied morpho dynamic parameters of embryos from aged and young NJ/C57BL6 rats by constant imaging. Raymahapatra et al. [14] proposed the significance of the embryologic study in IVF development and the possible part of AI, especially in DL methods, while improving this process characteristic. ART techniques assist in tackling infertility problems in couples through different medicinal proceedings. IVF is a method where the sperm and eggs are united beyond the body, allowing for fertilization and initial embryo growth in an organized atmosphere. The positive result of ART techniques, particularly IVF, depends on the embryo’s morphology and quality.

Einy et al. [15] proposed a new DL technique to differentiate the synchronous irregularity of embryos in TL methods for identifying non-live and live childbirths in IVF. The technique is implemented using a long short-term memory (LSTM) and local binary CNNs (LBCNNs). The LBCNNs enhance the identification precision by using local and deep feature groups with the smallest amount of learning parameters in contrast with a typical convolutional layer. Sharma et al. [16] presented AI methods to predict the kinetics of embryo morphologies during a term in the future. This AI method can be used to study the development of an embryo in the last 2 h and predict the morphological variations of the embryo for the succeeding 2 h. It uses a prediction method of combining convolutional LSTM layers to predict the impending video framed by studying the preceding morphologic variations in the sequence of the embryo’s video. Weatherbee et al. [17] proposed to study the signal connections after implantation utilizing the epiblast, hypoblast, and human embryo stem cell models. The authors determined that the forward hypoblast condition is NODAL-dependent in mice. Although BMP prevents forward signal center conditions in mice, it is vital for human preservation. Also, the different needs for BMP in the simple early developmental epiblast of human embryos and mice were identified. Zhang et al. [18] proposed widely examining the dynamical lipid landscapes in the initial development of human and mouse embryos. The lipid signs of various developing phases differed, especially for the phospholipid groups. The authors emphasized that more phospholipid unsaturation is preserved as embryos reach the blastocyst phase. Lipid desaturases like SCD1 were also needed for blastocyst implantation and in vitro blastocyst growth.

Singh et al. [19] emphasized the requirement for advanced risk evaluation methods for novel chemicals and nanomaterials, incorporating recent developments in quantitative structure–activity relationships (QSAR) with ML and computational modeling. It accentuates the role of nanodescriptors and diverse computational methods to improve predictive accuracy. Sarker et al. [20] presented COMFormer, a DL method for classifying maternal–fetal and brain anatomical structures in 2-D fetal ultrasound images. By implementing a transformer-based method with a novel residual cross-variance attention block, COMFormer effectually captures spatial and global features for precise classification of diverse anatomical categories. Yang et al. [21] proposed a hierarchical online contrastive anomaly detection (HOCAD) method. This contribution comprises a coarse-to-fine approach to the enhanced localization of MCCs and an online learning methodology with two new anomaly scores to filter out non-MCC anomalies from single images efficiently. Zhao et al. [22] introduced the TransFSM methodology, a hybrid Transformer framework for fetal multi-anatomy segmentation and biometric measurements. It employs deformable self-attention for multi-scale processing, a boundary-aware decoder for improved local detail, and an auxiliary segment head to enhance mask prediction and semantic correspondence among pixel categories. Sindhu and Annamalai [23] employed advanced DL methods, specifically vision transformers, to automate the detection of standard fetal ultrasound planes. Tang et al. [24] designed a two-stage ensemble learning approach based on sonography, Fgds-EL, to detect genetic diseases. Degala et al. [25] introduced the Attention Gate Double U-Net with a Guided Decoder (ADU-GD) model specifically crafted for fetal biometric parameter prediction. Liu et al. [26] proposed a label density-weighted loss with ranking similarity regularization (LDW-RS) method for deep imbalanced regression of fetal brain age. This loss captures the similarity between neighboring samples in the label space, enhancing model performance in imbalanced scenarios. Li et al. [27] developed FHUSP-NET, a DL network that automatically recognizes five fetal heart ultrasound planes (FHUSPs) and detects key anatomical structures. Employing spatial pyramid pooling and squeeze-and-excitation networks enhances feature representation and sensitivity while presenting an effectual IOU loss function for better similarity optimization.

The existing medical consent-tractable DL method for embryo segmentation and viability analysis may depend heavily on high-quality optical images, affecting accuracy in clinical settings. Focusing on the morula phase could overlook critical earlier developmental stages, limiting comprehensive analysis. Additionally, the usage of ML and time-lapse microscopy might be constrained by the availability of quality imaging data and may need to generalize better across species. Furthermore, while AI approaches exhibit promise in enhancing IVF outputs, their efficiency may vary in various clinical scenarios due to insufficient empirical validation. Methods depending on LSTM and CNNs might face difficulty with intrinsic temporal relationships and intrinsic uncertainties in embryo development. Also, findings from animal studies may only partially translate to human embryos, limiting applicability. The proposed risk evaluation methodologies for novel chemicals may also encounter threats in real-world applications, affecting predictive accuracy. Lastly, frameworks designed for fetal ultrasound detection might experience difficulties with various imaging conditions and patient populations, underscoring the requirement for robust, generalizable models. There is a notable gap in applying advanced ML methods for automated fetal ultrasound analysis, specifically in addressing variability across diverse imaging conditions and populations. Additionally, existing approaches often lack robust validation in real-world clinical scenarios, emphasizing the requirement for enhanced techniques that can generalize effectually to improve embryo analysis and monitoring outputs.

## 3. Proposed Method

**Figure 1:**
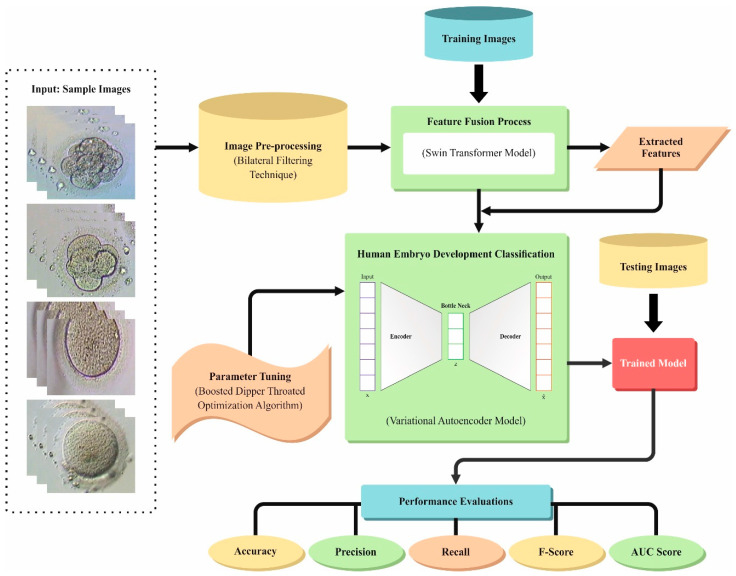
Overall process of EDMCV-STBDTO model.

In this article, a novel EDMCV-STBDTO method is introduced. The EDMCV-STBDTO method lies in accurate and efficient anomaly detection in embryo development and is critical for improving fertility treatments and advancing developmental biology using medical CV techniques. It includes distinct processes such as image preprocessing, feature extraction, classification-based VAE, and BDTO-based parameter tuning, as demonstrated in Figure 1.

### 3.1. Noise Reduction

**Figure 2:**
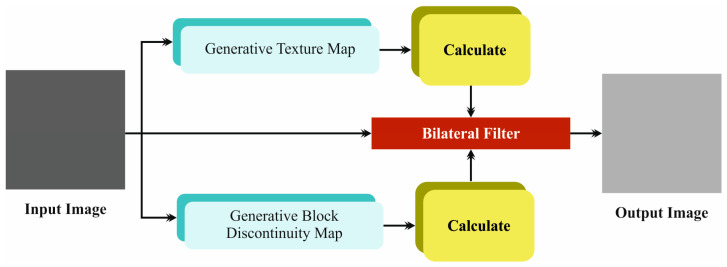
Structure of *BF* model.

Primarily, the EDMCV-STBDTO method performs image preprocessing using *BF* to remove the noise [28]. This technique is advantageous as it maintains crucial structural features in the images, unlike conventional filtering models that may blur edges. The *BF* model mitigates noise by selectively smoothing the image without compromising the data quality, which is crucial for additional evaluation. Furthermore, the *BF* technique is computationally efficient, making it appropriate for real-time applications. Its capability to adaptively adjust based on local pixel intensity discrepancies improves robustness against diverse noise levels. Overall, utilizing the *BF* model in the EDMCV-STBDTO method confirms high-quality input for subsequent processing steps, enhancing detection accuracy and reliability. Figure 2 depicts the architecture of the *BF* technique.

A *BF* smooths images and decreases noise while maintaining edges. Gaussian blurring is stated mathematically as follows:

(1) $$GB{\left[I\right]}_{p}=\sum_{q\in S}\;G_{o}\left(\left|\left|p=q\right|\right|I_{q}\right)$$

The result *GB* ${\left[I\right]}_{p}$ at pixel p is demonstrated directly above, and the RHS successfully consists of all pixels q weighted by the Gaussian model representing pixel q ’s intensity. The *BF* can be described as follows:

(2) $$BF\;[I]\;p=\frac{1}{W_{p}}\sum_{q\in S}\;G\sigma_{s}\left(\left|\left|p-q\right|\right||G|o_{r}|\left(\left|I_{p}-I_{q}\right|I_{q}\right)\right)$$

where $\frac{1}{W_{p}}$ specifies the normalization element, $Go_{r}\left(\left|I_{p}-I\right|\right)$ characterizes range weight, and $\left(Go_{s}\left|\left|p-q\right|\right|\right)$ means space weight.

In these instances, the range weight and normalization factor are added terms in the previous equation. $\sigma_{s}$ refers to the spatial degree of the kernels, for example, the neighborhood size, and $\sigma_{r}$ represents a lower amplitude of edges. It promises that single pixels with intensity levels equal to the center pixel are calculated to be blurry while keeping sharper intensity fluctuations. The sharper the edge, the lesser the value of $\sigma_{r}$ . This equation results in a Gaussian blur $\sigma_{r}$ by approaching infinities.

### 3.2. Feature Extraction Using Swin Transformation

**Figure 3:**
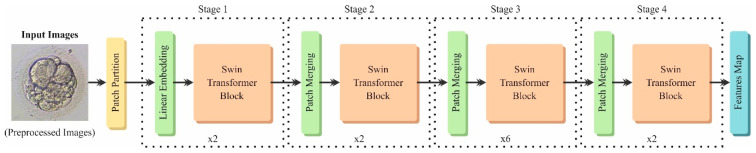
Framework of ST model.

Next, the ST method is utilized for the feature extraction method [29]. The feature extraction process using the ST model is a compelling choice due to its capability to capture local and global contextual data through a hierarchical representation. Unlike conventional CNNs, which may face difficulty with complex spatial relationships, the ST model utilizes a shifted windowing mechanism that allows for effectual processing of images at varying scales. This approach improves the capacity of the technique to understand complex patterns and structures within the data, making it specifically efficient for tasks in medical imaging and anomaly detection. Moreover, the ST technique performs better on benchmark datasets, underscoring its robustness and adaptability across various applications. Its integration of self-attention mechanisms enhances feature relevance, confirming that the most informative aspects of the data are prioritized for evaluation. Overall, the ST approach provides a modern, efficient methodology for feature extraction that substantially improves the model’s performance. Figure 3 illustrates the ST model.

The presented swin transformer structure applied in this study is a hierarchical Vision Transformer intended for numerous vision tasks. The structure uses various new models to process and transform input data proficiently.

The swin Transformer initially splits an input image into independent sections using a patch-splitting module, equivalent to the Vision Transformer (ViT). Every patch is processed as a segmentation, and its features are concatenated over the numerous layers in the network. For instance, assuming a patch size of 8×8 , the feature size of every section should be 8×8×3=192 . A layer of linear embedding then designs this natural feature into a random dimensionality C.

#### 3.2.1. Phase 1: Early Transformation and Embedding

This patch utilizes numerous transformer blocks adapted to contain swin transformer blocks with shifted window-based self-attention modules (SW-MSA). It features mapping segmentations. The early embedding keeps the amount of segmentation, which can be signified as $\frac{H}{4}\times \frac{W}{4}$ .

#### 3.2.2. Phase 2: Hierarchical Representation

The hierarchical representation is attained to reduce the segmentations by the layers of patch-merging as the network increases. The initial layer of patch-merging chains is the feature of every collection of 2×2 nearby segments, and it uses a linear layer to the 4C -dimension chained feature mappings. These assist in decreasing the number of segmentations by a feature of a quartet, downsampling the resolution to 2×2 . The output dimensionality is set to 2*C*. After this, the transformer blocks are utilized for the feature mappings while maintaining the resolution at $\frac{H}{8}\times \frac{W}{8}.$

#### 3.2.3. Phases 3 and 4: Additional Hierarchical Representation

The process is reiterated for phases 3 and 4, corresponding to without-put resolutions of $\frac{H}{16}\times \frac{W}{16}$ and $\frac{H}{32}\times \frac{W}{32}$ . Every phase gradually decreases the number of segmented feature mappings and upsurges the feature dimensionality, offering a hierarchical representation similar to conventional convolutional networks such as ResNet, VGG, and ViT.

In the network phases, a swing transformer block substitutes the standard multi-head self-attention (MSA) module with a shifted window-based MSA module (SW-MSA) block. Every swing transformer block contains the subsequent modules.

- SW-MSA.
- A dual-layer function of multilayer perceptron (*MLP*) with Gaussian Error Linear Unit (GELU).
- Normalization layers (LNs) are used before every MSA and *MLP* element.
- Residual connections are used next to every module.

Due to SW-MSA, an input feature mapping $f\in R^{H\times W\times C}$ . The attention operation inside a segmentation is expressed as Equation (3):

(3) $$Att\left(Q,\;K,\;V\right)=SoftMax\left(\frac{QK^{T}}{\sqrt{d_{k}}}\right)V$$

*V*, *Q*, and *K* represent the corresponding value, query, and key matrices. The key dimension is $d_{k}$ . The output of every swin transformer block is formulated in Equation (4).

(4) $$O=MLP\left(LN\left(Att\left(LN\left(f\right)\right)\right)\right)$$

### 3.3. Classification Using VAE Model

**Figure 4:**
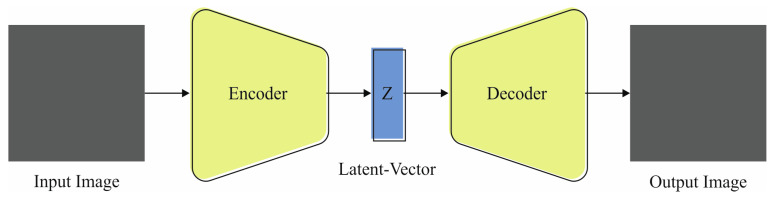
Architecture of VAE technique.

For the classification of human embryo development, the EDMCV-STBDTO model applies the VAE method [30]. The VAE model is ideal for classification tasks because it can effectively capture intrinsic data dispersions through latent variable modeling. Unlike conventional autoencoders, VAEs provide a probabilistic method that allows for the generation of new data samples, improving robustness and flexibility in classification. This generative capability assists in learning more representative features, making the model resilient to discrepancies in the input data. Moreover, VAEs integrate regularization through a *KL* divergence term, which promotes better generalization and mitigates overfitting compared to other methodologies. Their capability to handle missing data and perform semi-supervised learning distinguishes VAEs as a powerful tool in various applications comprising image and speech recognition, where capturing underlying patterns is significant for efficient classification. Figure 4 depicts the architecture of the VAE model.

It contains decoding and encoding systems, where the latent space can be normalized to encode every model, such as distribution across the latent space. Equated to AE, the VAE latent space can be constant and easy to incorporate. Rather than studying a latent space, VAE studies a distribution of the latent space, out of which latent vectors are tested. VAE enforces a particular framework in the latent space and assures intercalations during the latent space, enabling reliable renovations. Unlike AE, VAE has creative capability; specifically, VAE could create a novel sample that needs to be included in the input.

In the training of VAE, the encoding creates vectors of μ and log -variance $\left(\sigma^{2}\right)$ . Later, a vector $\underline{z}$ can be taken from N (0, 1) and resized to create the latent vector $z=\mu +\sigma .\underline{z}$ that drives the decoding to create the recreated input data $\underline{x}$ . Furthermore, the divergence of *KL* makes μ and σ to become 0 and 1 correspondingly. One main concern of VAE is whether it is vulnerable to later failure, in which the tested z can be weak. $\underline{x}$ becomes nearly independent of z .

In VAE, the decoding calculates the restricted probability distribution $P_{\theta }(x|z)$ , whereas the encoding calculates the estimated latter distribution $Q_{\emptyset }(z|x)\approx P_{\theta }(z|x)$ . In VAE, data can be utilized to enhance the parameters of decoding θ to decrease the reconstructing error and the encoding parameters ∅ to create $Q_{\emptyset }(z|x)$ as adjacent as possible to the latter distribution $P_{\theta }(z|x)$ . The word regularization utilizes the divergence of Kullback–Liebler (*KL*), which determines how near the encoder latent vector distribution $Q_{\emptyset }(z|x)$ is to the estimated posteriors of the preceding distribution $P_{\theta }(z)$ anticipated over a usual Gaussian. The overall loss to be reduced is stated based on *KL* divergence.

(5) $$D_{KL}(Q_{\emptyset }(z|x)\|P_{\theta }(z|x))=E_{Z\sim Q}\left[logQ_{\emptyset }\left(x\right)-logP_{\theta }\left(x\right)\right]$$

$P_{\theta }(z|x)$ denotes the latter distribution, for example. The encoding vector distribution provides the decoding one. Applying Baye’s theorem on $P_{\theta }(z|x)$ , Equation (5) becomes

(6) $$-D_{KL}(Q_{\emptyset }(z|x)\|P_{\theta }(z|x))=-E_{Z∼Q}[logQ_{\emptyset }(z|x)-logP_{\theta }(z)]\;+E_{Z∼Q}\left[logP_{\theta }\left(z\right)\right]-logP_{\theta }\left(x\right)$$

It is further rewritten as

(7) $$log\;P_{\theta }(x)-D_{KL}(Q_{\emptyset }(z|x)\|P_{\theta }(z|x))=E_{Z∼Q}[logP_{\theta }(x|z)]\;-D_{\zeta L}(Q_{\emptyset }(z|x)\;\left\|P_{\theta }\left(z\right)\right)$$

The VAE loss function contains an unambiguous format, destructive of evidence lower bound (ELBO), as presented on the right side of the equations. The initial portion can be a loss of reconstruction that increases the log probability of the latter distribution. The second portion represents a regularization word that reduces the encoding distribution and the preceding latent distribution so that the encoding studies concentrate on the previous. The optimizer aims to enhance the reconstruction and reduce the divergence of the *KL* between the approximated one and the real posterior. The initial expression on the right side of the formula denotes the negative, while $P_{\theta }(x|z)$ can be presumed to be a Gaussian distribution. The greatest estimation of the encoding is attained when $D_{KL}(Q_{\emptyset }(z|x)\|P_{\theta }(z))$ is nearly 0.

### 3.4. BDTO-Based Parameter Tuning

**Figure 5:**
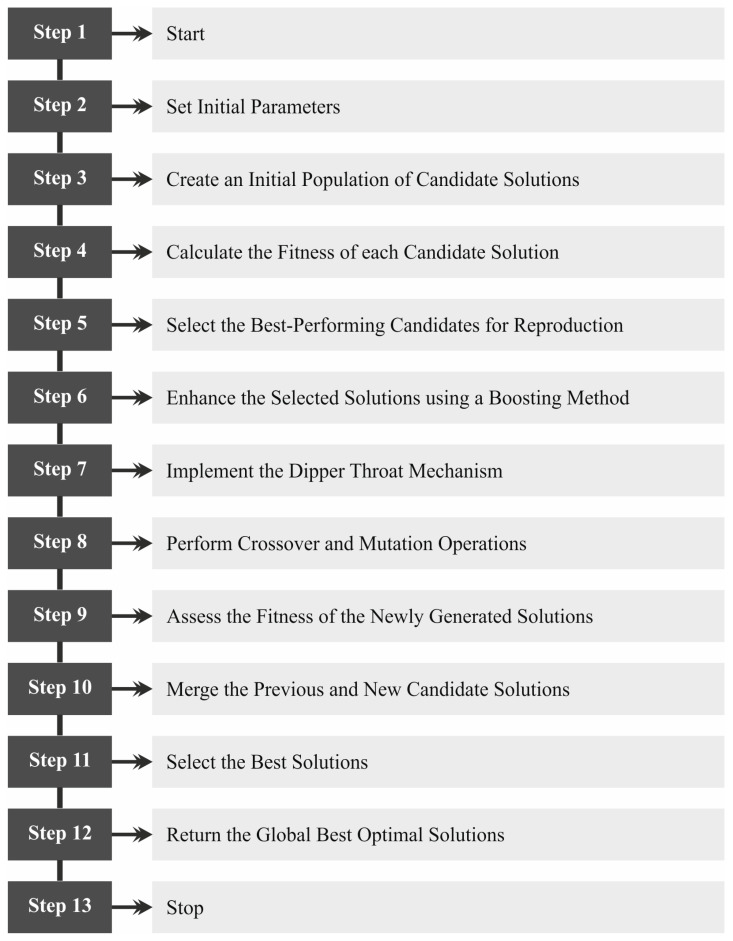
Workflow of BDTO approach.

Finally, the hyperparameter selection of the VAE technique is implemented by the design of the BDTO model [31]. This technique is an effective parameter-tuning methodology because it balances exploration and exploitation in search spaces. By improving the original DTO model, the BDTO method incorporates adaptive mechanisms that alter the search strategy based on the landscape of the objective function, resulting in more effective convergence. This methodology is advantageous in intrinsic and high-dimensional parameter spaces, where conventional optimization models may encounter difficulty. Moreover, the capability of the BDTO model to escape local optima and maintain diversity in the search process assists in ensuring that the most promising regions of the parameter space are explored thoroughly. Overall, its robustness, adaptability, and efficient integration make BDTO ideal for optimizing model parameters in diverse applications. Figure 5 depicts the workflow of the BDTO model.

The dipper-throated bird is considered an organ kind of Cinclus within the Cinclidae bird group because of its stirring down and up or dripping gestures. Its ability to hunt, dive, and swim on the sea bottom is typical of other birds. Additionally, as it retains smaller and flexible wings, it can acquire them directly and rapidly without interruptions and move easily.

A dipper-throated bird keeps its excellent searching model; it achieves quick, flexible activities and is improved at the breast-clean white level. When the prey can be determined, it primarily puts its head into the water, even into the wild water, and flows quickly. When it reaches the bottom, it uplifts stones and causes disturbances to irritate sea creatures, sea animals, and small fishes.

The specific steps on the bottom water levels with grasping stones involve the bird regularly stepping opposite the current time whereas its head can be placed down to find the objective; it is balanced with its stronger feet for an extended period; additionally, it can step into the sea and purposefully swim with its wings effectively and step by the end dropping its head and body at an angle to find the food.

The DTO method considers that people fly and swim to hunt food bases. The values of $N_{fs}$ are accessible for candidate n . The location (P) and velocity of the candidate (V) are expressed as Equations (8) and (9):

(8) $$P=\left[P_{1,1}\;P_{1,2}\;P_{1,3}\;\cdots \;P_{1,d}\;P_{2,1}\;P_{2,2}\;P_{2,3}\;\cdots \;P_{2,d}\;P_{3,1}\;P_{3,2}\;P_{3,3}\;\cdots \;P_{3,d}\;\cdots \;\cdots \;\cdots \;\cdots \;\cdots \;P_{n,1}\;P_{n,2}\;P_{n,3}\;\cdots \;P_{n,d}\right]$$

(9) $$V=\left[V_{1,1}\;V_{1,2}\;V_{1,3}\;\cdots \;V_{1,d}\;V_{2,1}\;V_{2,2}\;V_{2,3}\;\cdots \;V_{2,d}\;V_{3,1}\;V_{3,2}\;V_{3,3}\;\cdots \;V_{3,d}\;\cdots \;\cdots \;\cdots \;\cdots \;\cdots \;V_{n,1}\;V_{n,2}\;V_{n,3}\;\cdots \;V_{n,d}\right]$$

$P_{i,j}$ describes bird i in measurement j while i∈1,2,3, …, n and j∈1,2,3, …, $d.\;V_{ij}$ represents the individual velocity i in measurement j for i∈1,2,3, …, n and j∈1,2,3, …, d . The key positions of $P_{i,j}$ are frequently spread at high and lower limits. The *fitness* values $f=f1,f2,f3,\ldots f_{n}$ are intended for each bird as in the array in Equation (10):

(10) $$P=\left[f_{1}(P_{1,1}\;P_{1,2}\;P_{1,3}\;\cdots \;P_{1,d})\;f_{2}(P_{2,1}\;P_{2,2}\;P_{2,3}\;\cdots \;P_{2,d}\;f_{3}(P_{3,1}\;P_{3,2}\;P_{3,3}\;\cdots \;P_{3,d}\;\cdots \;\cdots \;\cdots \;\cdots \;f_{n}(P_{n,1}\;P_{n,2}\;P_{n,3}\;\cdots \;P_{n,d}\right]$$

Now, the cost value defines the food resource qualities observed by all birds. The mother bird can be described as the optimum value. Later, these quantities are structured in increasing order. The first highest solution is set to $P_{best}$ . The residual solutions are viewed as typical individuals $P_{nd}$ of supporting ones. The highest global solution is considered $P_{Gbest}.$

The DTO technique of the present optimizer methods for restarting the swimming candidate condition was initiated to be in understanding using Equation (11):

(11) $$Pnd\left(t+1\right)=Pgreatest(t)-S1.|S2.Pgreatest(t)-Pnd(t)$$

Pnd(t) represents a usual bird’s location at iteration t , and Pgreatest (t) is regarded as the best candidate location. “.”is considered the pairwise multiplication. Pnd(t+1) is initiated to restore the individual area of the solution. S1 and S2 are altered in the iteration, which is presented as Equation (12):

(12) $$1=2s.r1-s\;S2=2r1\;s=2(1-(\frac{t}{T_{max}}{)}^{2})$$

whereas s differs exponentially from [2–0], r1 denotes a stochastic quantity among [ 0 , 1], and $T_{max}$ denotes the greatest number of iterations. The second mechanism of the method mentioned above is regarded as the source of improving the velocity of the individual and positions over the following Equation (13):

(13) $$Pnd\left(t+1\right)=Pnd\left(t\right)+V\left(t+1\right)$$

where $P_{nd}(t+1)$ is considered the novel individual location of typical candidates, and each revival velocity V(t+1) is computed using Equation (14):

(14) $$V\left(t+1\right)=S3V\left(t\right)+S4r2\left(Pgreatest\left(t\right)-Pnd\left(t\right)\right)\;+S5r2\left(PGgreatest-Pnd\left(t\right)\right)$$

S3 denotes a weighted value, S4 and S5 are coefficients, PGgreatest represents the global best location, and r2 represents a stochastic volume in [0, 1].

The DTO method is selected by Equation (15):

(15) Pnd(t+1)={Pgreatest(t)−S1.|M| if R<0.5 Pnd(t)+BV(t+1) otherwise

Now, M=S2.Pbest(t)−Pnd(t) and R are stochastic volumes in [0–1].

The DTO method requires alteration to improve its execution and efficiently tackle the optimizer problems. The primary method can have limits or features that are developed. Over the execution of changes, the purpose is to enhance its converging speed, search efficacy, and complete qualities of the solution.

The changes, called the BDTO method, involve active changes to the word R that indicate the distinct locations in the method. The dynamic nature of this alteration qualifies as flexibility and adaptability during the optimization process. Subsequently, the value of R undergoes modifications in the process of optimization. These dynamic modifications could contain numerous differences, like arbitrary individual changes, adaptable modifications based on *fitness* values, or other tactics intended to present exploration and diversity. The changes considered in this context enhance the equality between exploration and exploitation in the method. This alteration enables a more inclusive search space exploration while efficiently developing guaranteeing areas. By dynamically changing the variable R , the process becomes increasingly efficient in evading local optimum and finding greater solutions.

By executing the BDTO method, it is predicted that numerous advantages should be perceived. These include an improved convergence speed, enhanced exploitation and exploration abilities, and, eventually, a more excellent inclusive execution in resolving the optimizer issues. Rather than allocating a stable value to R , handling it as an adjustable represented as Rω that is dynamically modified at every iteration is recommended. The solitary potential technique to attain this is using a reduction function depending on the number of iterations. Equation (16) combines this development:

(16) $$R_{j}=R_{max}\times exp\left(-\alpha \times \frac{i}{{max}_{iter}}\right)$$

At the present iteration i, $R_{i}$ denotes the ongoing value. At the same time, the maximal value of R , a scaling factor α , which identifies the reduction rate, and the uppermost number of iterations probable, are represented by the variable $ma_{xter}$ . The method uses a dynamic scaling factor to gradually reduce the R -value since it ensues over the following iteration. This feature improves the method’s capability to utilize the optimizer process in the advanced phases, although it also enables better exploration in the initial stages.

The BDTO model uses a *fitness* function (FF) to reach better-performing classification. It decides a positive integer to indicate the superior performance of the solutions candidate. In this work, the reduction of the *classification error rate* can be determined for the FF, as expressed in Equation (17).

(17) $$fitness\left(x_{i}\right)=ClassifierErrorRate\left(x_{i}\right)\;=\frac{no\;of\;misclassified\;samples}{Total\;no\;of\;samples}\times 100$$

## 4. Experimental Validation

**Figure 6:**
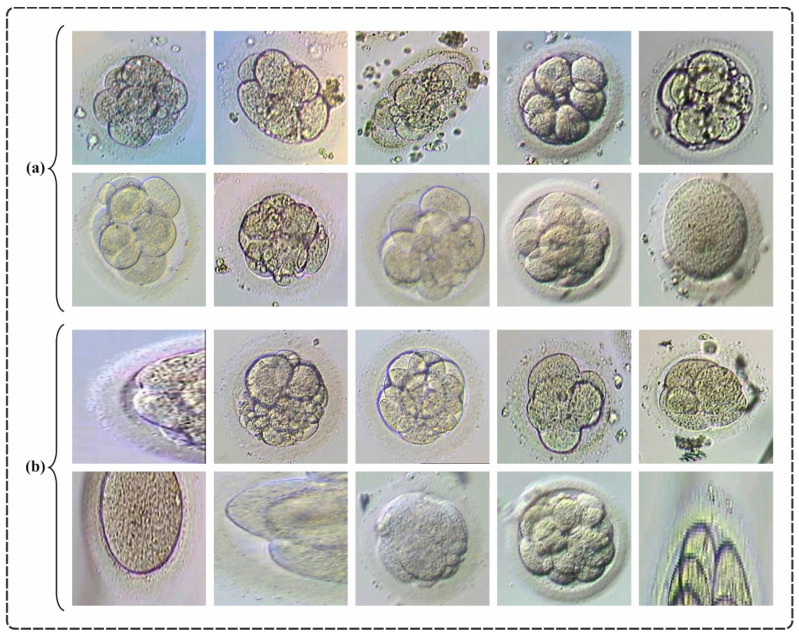
Sample images: (**a**) Good and (**b**) Not-Good.

In this section, the performance analysis of the EDMCV-STBDTO model is examined under the Kaggle dataset [32], which comprises significant components for a DL competition, featuring training and testing folders with images of day-3 and day-5 embryos, along with CSV files: train.csv, test.csv, and sample_submission.csv. Images are saved in JPG format, labeled with prefixes (D3 for day 3 and D5 for day 5) for easy detection. The goal is to create a technique that classifies embryo images as 1 (good) or 0 (not good). Every image is assigned an ID, and the final submission file should contain two columns: ID and Class. The columns of the dataset encompass ID, Image, and Class, with the latter indicating the ground truth label for embryo quality. Exploring and preprocessing the data are significant for building an efficient classification technique. The dataset has 620 images of two classes, as presented in Table 1. Figure 6 demonstrates the sampled images. The suggested technique was simulated using the Python 3.6.5 tool on PC i5-8600k, 250 GB SSD, GeForce 1050Ti 4 GB, 16 GB RAM, and 1 TB HDD. The parameter settings are provided as follows: learning rate, 0.01; activation, ReLU; epoch count, 50; dropout, 0.5; and batch size, 5.

**Figure 7:**
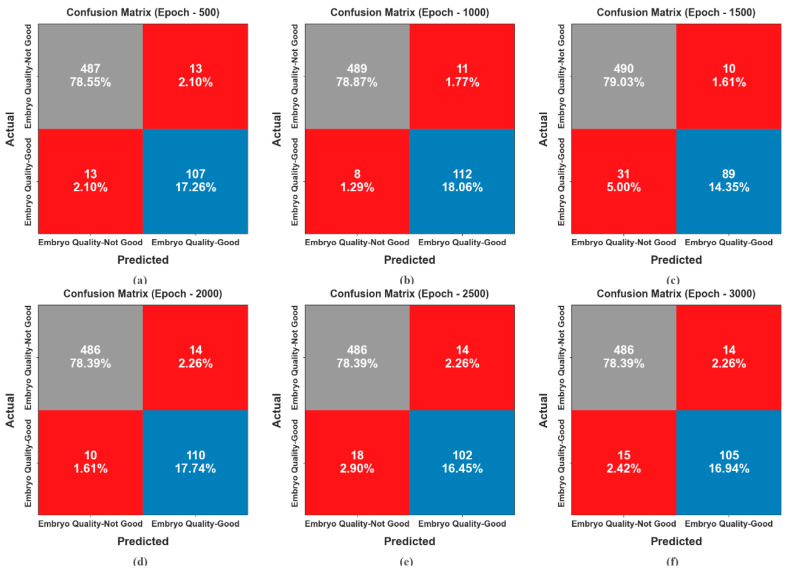
Confusion matrices of EDMCV-STBDTO technique: (**a**–**f**) Epochs 500–3000.

Figure 7 shows the confusion matrices created by the EDMCV-STBDTO approach under various epoch counts. The findings indicate that the EDMCV-STBDTO methodology contains effective, accurate recognition of all three classes. The confusion matrices for various epochs exhibit the model’s performance in classifying embryo quality. At epoch 500, the model attained 487 correct predictions for good-quality embryos, while 78.55% of the predictions were accurate overall. At epoch 1000, the accuracy enhanced slightly to 489 correct predictions and a 78.87% overall accuracy. By epoch 1500, the model reached 490 correct predictions, providing a 79.03% accuracy rate. However, by epoch 2000, the performance lessened slightly to 486 correct forecasts with an accuracy of 78.39%. Finally, at epoch 2500, the model reached 486 correct predictions again and maintained a 78.39% accuracy. The results demonstrate fluctuations in performance across epochs, emphasizing the need for additional optimization.

**Figure 8:**
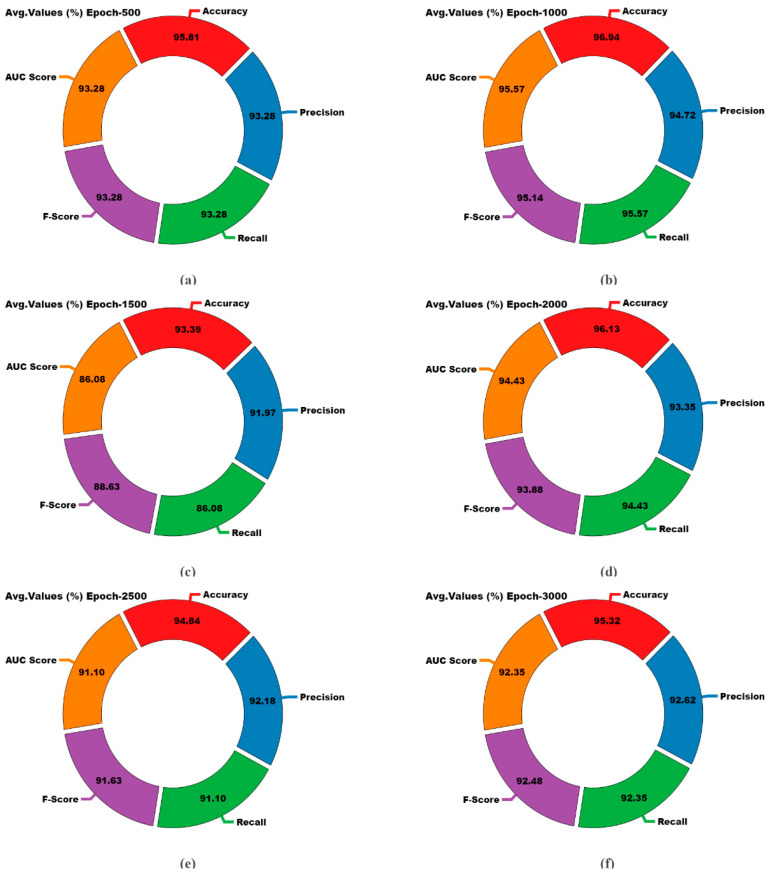
Average outcome of EDMCV-STBDTO technique: (**a**–**f**) Epochs 500–3000.

The classifier results of the EDMCV-STBDTO methodology are presented for different numbers of epochs in Table 2 and Figure 8. The table’s values show that the EDMCV-STBDTO methodology accurately recognized all the classes. On a 500 epoch count, the EDMCV-STBDTO method had an average $accu_{y}$ of 95.81%, $prec_{n}$ of 93.28%, $reca_{l}$ of 93.28%, $F_{score}$ of 93.28%, and ${AUC}_{score}$ of 93.28%. Moreover, on a 1000 epoch count, the EDMCV-STBDTO method had an average $accu_{y}$ of 96.94%, $prec_{n}$ of 94.72%, $reca_{l}$ of 95.57%, $F_{score}$ of 95.14%, and ${AUC}_{score}$ of 95.57%. Additionally, on a 1500 epoch count, the EDMCV-STBDTO approach had an average $accu_{y}$ of 93.39%, $prec_{n}$ of 91.97%, $reca_{l}$ of 86.08%, $F_{score}$ of 88.63%, and ${AUC}_{score}$ of 86.08%. Similarly, on a 3000 epoch count, the EDMCV-STBDTO approach had an average $accu_{y}$ of 95.32%, $prec_{n}$ of 92.62%, $reca_{l}$ of 92.35%, $F_{score}$ of 92.48%, and ${AUC}_{score}$ of 92.35%.

**Figure 9:**
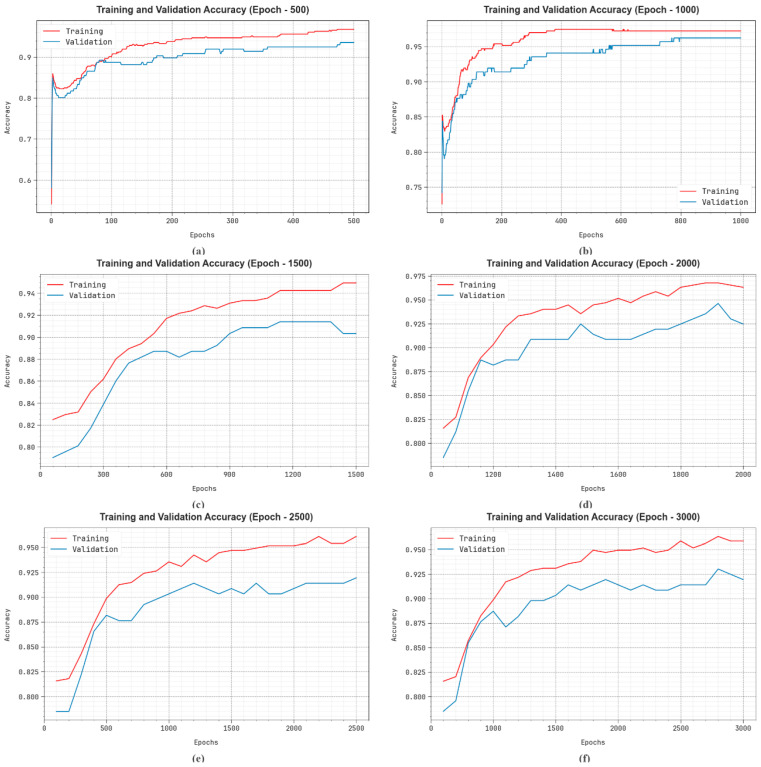
$Accu_{y}$ curve of EDMCV-STBDTO technique: (**a**–**f**) Epochs 500–3000.

In Figure 9, the training $Accu_{y}$ (TRAAC) and validation $Accu_{y}$ (VLAAC) outcomes of the EDMCV-STBDTO method at different numbers of epochs are stated. The $Accu_{y}$ results are estimated for 0–3000 epochs. The figure shows that the TRAAC and VLAAC values exhibit a rising tendency that informs the ability of the EDMCV-STBDTO technique to deliver superior outcomes across various numbers of iterations. Furthermore, the TRAAC and VLAAC stay adjacent across the epochs, which defines less minimum overfitting and displays improved results of the EDMCV-STBDTO technique, promising a constant prediction on unidentified samples.

**Figure 10:**
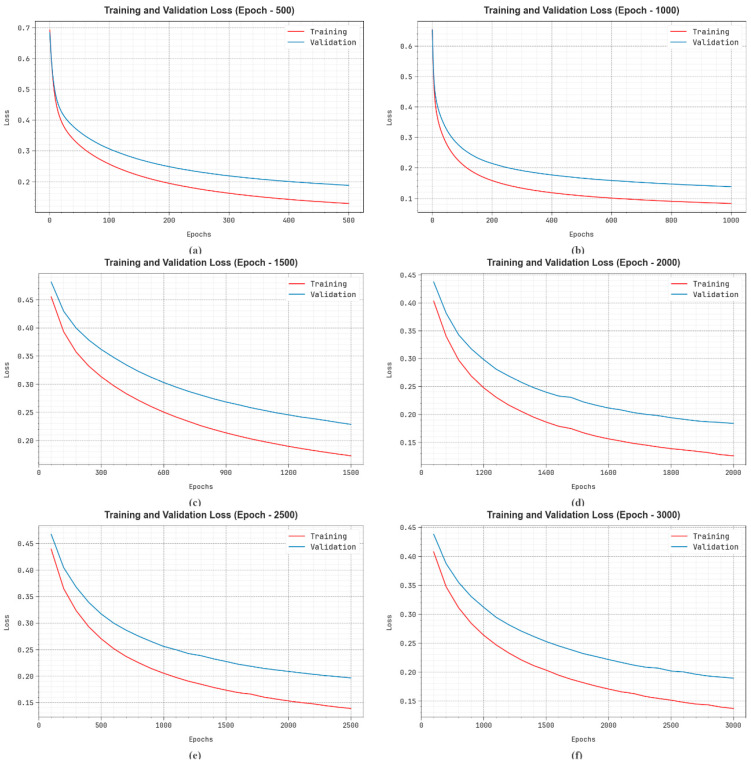
Loss curve of EDMCV-STBDTO technique: (**a**–**f**) Epochs 500–3000.

In Figure 10, the TRA loss (TRALS) and VLA loss (VLALS) graph of the EDMCV-STBDTO technique is represented in terms of various numbers of epochs. The loss values are estimated throughout 0–3000 epoch counts. It is demonstrated that the TRALS and VLALS values describe a lowering trend, indicating the capability of the EDMCV-STBDTO approach to balance a trade-off between data fitting and generalization. The continual reduction in loss values also promises better results for the EDMCV-STBDTO approach and tuning of the prediction values in time.

**Figure 11:**
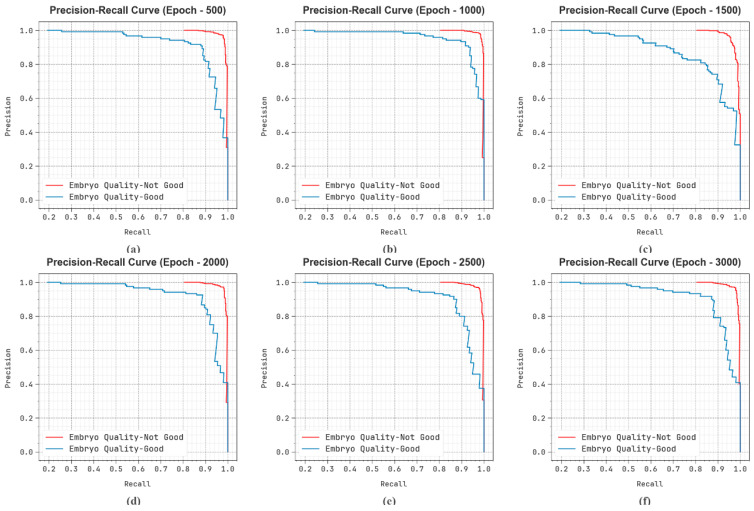
PR curve of EDMCV-STBDTO technique: (**a**–**f**) Epochs 500–3000.

In Figure 11, the precision–recall (PR) investigation study of the EDMCV-STBDTO technique on different numbers of epochs provides an interpretation of its outcomes by plotting precision against recall for each class label. The figure shows that the EDMCV-STBDTO technique repeatedly achieves enhanced PR values through several classes, portraying its ability to preserve an important segment of true positive predictions between each positive prediction (precision) but additionally taking a larger amount of actual positives (recall). The continuous growth in PR values between all class labels demonstrates proficiency in the EDMCV-STBDTO method in the classification process.

**Figure 12:**
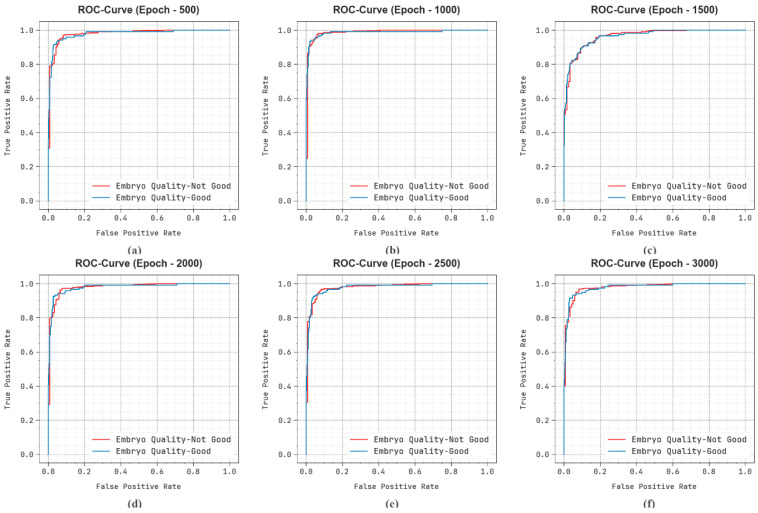
ROC curve of EDMCV-STBDTO technique: (**a**–**f**) Epochs 500–3000.

Figure 12 shows the ROC examination of the EDMCV-STBDTO methodology on the number of epochs. The findings illustrate that the EDMCV-STBDTO approach obtains increased ROC values across every class, depicting important capability of differentiating the class labels. This steady tendency of enhanced ROC outcomes on several class labels indicates the efficient results of the EDMCV-STBDTO approach in predicting different numbers of classes, showing the robust nature of the classifier method.

**Figure 13:**
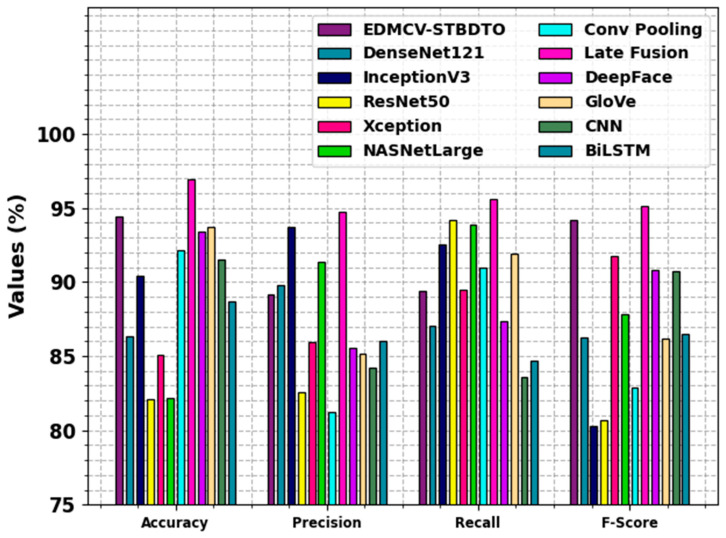
Comparative analysis of EDMCV-STBDTO technique with recent methods.

In Table 3 and Figure 13, the stimulated values of the EDMCV-STBDTO approach are consistent with contemporary works [33,34,35,36,37]. The EDMCV-STBDTO approach achieved superior results in diverse value assessment methods. The performance comparison of diverse techniques shows notable differences in accuracy, precision, recall, and F1 scores. The EDMCV-STBDTO model attained an accuracy of 94.42, with a precision of 89.15, recall of 89.37, and F1 score of 94.18, outperforming others such as DenseNet121 (accuracy: 86.31), InceptionV3 (accuracy: 90.42), and ResNet50 (accuracy: 82.11). Late Fusion emerged as the top performer, with an accuracy of 96.94 and an F1 score of 95.14. Other methods, comprising Xception, NASNetLarge, and BiLSTM, portrayed varying degrees of efficiency, with metrics demonstrating the significance of model selection depending on specific classification requirements.

Compared with $accu_{y}$ , the EDMCV-STBDTO approach shows its superiority with an improved $accu_{y}$ of 94.42%. At the same time, the DenseNet121, InceptionV3, ResNet50, Xception, NASNetLarge, Conv Pooling, and Late Fusion models achieved lower results with an $accu_{y}$ of 86.31%, 90.42%, 82.11%, 85.09%, 82.14%, 92.15%, and 96.94%, individually. Moreover, depending on $F_{score}$ , the EDMCV-STBDTO approach reached a better $F_{score}$ of 94.18%. In contrast, the DenseNet121, InceptionV3, ResNet50, Xception, NASNetLarge, Conv Pooling, and Late Fusion methods achieved reduced outcomes with an $F_{score}$ of 86.29%, 80.29%, 80.67%, 91.77%, 87.83%, 82.89%, and 95.14%, respectively.

**Figure 14:**
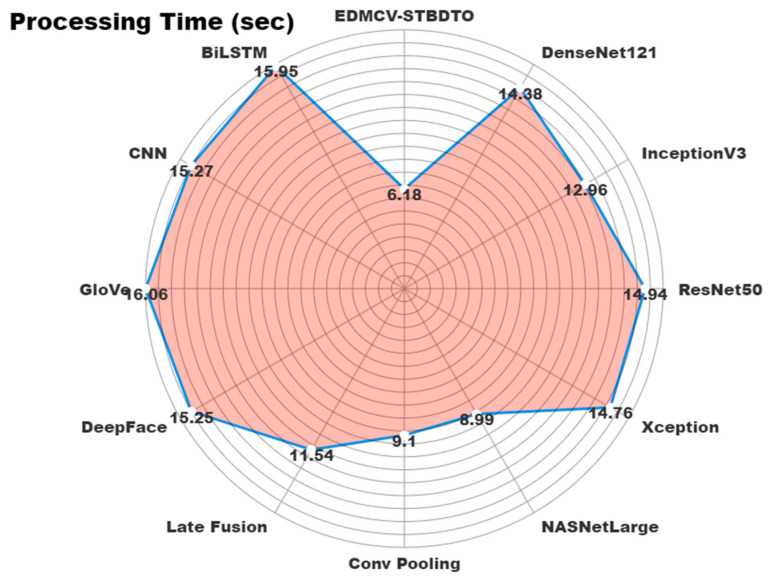
PT outcome of EDMCV-STBDTO technique with recent models.

In Table 4 and Figure 14, the comparison analysis of the EDMCV-STBDTO technique is demonstrated under processing time (PT). The values indicate that the EDMCV-STBDTO technique obtained superior outcomes. According to PT, the DenseNet121, InceptionV3, ResNet50, Xception, NASNetLarge, Conv Pooling, Late Fusion, DeepFace, GloVe, CNN, and BiLSTM models achieved better PT values of 14.38 s, 12.96 s, 14.94 s, 14.76 s, 8.99 s, 9.10 s, 11.54 s, 15.25 s, 16.06 s, 15.27 s, and 15.95 s while the EDMCV-STBDTO methodology attained a lower PT of 6.18 s.

## 5. Conclusions

In this article, a novel EDMCV-STBDTO technique is introduced. The EDMCV-STBDTO technique lies in accurate and efficient anomaly detection in embryo development and is critical for improving fertility treatments and advancing developmental biology using medical CV techniques. It includes distinct processes such as image preprocessing, feature extraction, classification-based VAE, and BDTO-based parameter tuning. Primarily, the EDMCV-STBDTO method performs image preprocessing using *BF* to remove the noise. Next, the swin Transformer method is used for the feature extraction technique. For the classification of human embryo development, the EDMCV-STBDTO method applies the VAE method. Finally, the hyperparameter selection of the VAE technique is implemented by the design of the BDTO model. The efficiency of the EDMCV-STBDTO method is validated by comprehensive studies using a benchmark dataset. The experimental validation of the EDMCV-STBDTO method showed that it attained a superior accuracy value of 94.42% over existing techniques. The EDMCV-STBDTO method’s limitations depend on the quality and diversity of the training data, which may hinder its capability to generalize across diverse embryo images. Furthermore, the complexity of the technique may result in longer training times and enhanced computational resource requirements. Its performance may also be affected by overfitting if not correctly regularized. Future work should focus on expanding the dataset to encompass a wide range of embryo stages and discrepancies to improve robustness. Employing more advanced ensemble methodologies could enhance classification accuracy. Moreover, integrating domain-specific knowledge from reproductive science may refine the model’s understanding and interpretation of embryo quality.

## Figures and Tables

**Table 1:** Details of dataset.

Classes	No. of Images
Embryo Quality-Not Good	500
Embryo Quality-Good	120
Total Images	620

**Table 2:** Classification outcome of EDMCV-STBDTO technique under distinct epochs.

Class	$Accu_{y}$	$Prec_{n}$	$Reca_{l}$	$F_{score}$	$AUC_{score}$
Epoch-500					
Embryo Quality-Not Good	95.81	97.40	97.40	97.40	93.28
Embryo Quality-Good	95.81	89.17	89.17	89.17	93.28
Average	95.81	93.28	93.28	93.28	93.28
Epoch-1000					
Embryo Quality-Not Good	96.94	98.39	97.80	98.09	95.57
Embryo Quality-Good	96.94	91.06	93.33	92.18	95.57
Average	96.94	94.72	95.57	95.14	95.57
Epoch-1500					
Embryo Quality-Not Good	93.39	94.05	98.00	95.98	86.08
Embryo Quality-Good	93.39	89.90	74.17	81.28	86.08
Average	93.39	91.97	86.08	88.63	86.08
Epoch-2000					
Embryo Quality-Not Good	96.13	97.98	97.20	97.59	94.43
Embryo Quality-Good	96.13	88.71	91.67	90.16	94.43
Average	96.13	93.35	94.43	93.88	94.43
Epoch-2500					
Embryo Quality-Not Good	94.84	96.43	97.20	96.81	91.10
Embryo Quality-Good	94.84	87.93	85.00	86.44	91.10
Average	94.84	92.18	91.10	91.63	91.10
Epoch-3000					
Embryo Quality-Not Good	95.32	97.01	97.20	97.10	92.35
Embryo Quality-Good	95.32	88.24	87.50	87.87	92.35
Average	95.32	92.62	92.35	92.48	92.35

**Table 3:** Comparative analysis of EDMCV-STBDTO technique with recent methods.

Methodology	$Accu_{y}$	$Prec_{n}$	$Reca_{l}$	$F_{Score}$
EDMCV-STBDTO	94.42	89.15	89.37	94.18
DenseNet121	86.31	89.78	87.06	86.29
InceptionV3	90.42	93.70	92.55	80.29
ResNet50	82.11	82.53	94.18	80.67
Xception	85.09	85.91	89.48	91.77
NASNetLarge	82.14	91.33	93.89	87.83
Conv Pooling	92.15	81.23	91.00	82.89
Late Fusion	96.94	94.72	95.57	95.14
DeepFace	93.37	85.53	87.36	90.82
GloVe	93.75	85.15	91.94	86.19
CNN	91.51	84.25	83.57	90.73
BiLSTM	88.67	86.03	84.70	86.51

**Table 4:** PT outcome of EDMCV-STBDTO technique with recent models.

Methodology	Processing Time (s)
EDMCV-STBDTO	6.18
DenseNet121	14.38
InceptionV3	12.96
ResNet50	14.94
Xception	14.76
NASNetLarge	8.99
Conv Pooling	9.10
Late Fusion	11.54
DeepFace	15.25
GloVe	16.06
CNN	15.27
BiLSTM	15.95

## Data Availability

The data supporting this study’s findings are openly available in the Kaggle repository at https://www.kaggle.com/competitions/world-championship-2023-embryo-classification/data (accessed on 12 May 2024), reference number [32].
